# Identifying Obstructive Hypertrophic Cardiomyopathy from Nonobstructive Hypertrophic Cardiomyopathy: Development and Validation of a Model Based on Electrocardiogram Features

**DOI:** 10.5334/gh.1250

**Published:** 2023-08-04

**Authors:** Lanyan Guo, Zhiling Ma, Weiping Yang, Fuyang Zhang, Hong Shao, Liwen Liu, Chao Gao, Ling Tao

**Affiliations:** 1 Department of Cardiology, Xijing Hospital, the Fourth Military Medical University, Xi’an, Shaanxi, China; 2 Department of Ultrasound, Xijing Hospital, the Fourth Military Medical University, Xi’an, Shaanxi, China; 3 Department of Cardiology, Radboud University Medical Center, Nijmegen, The Netherlands

**Keywords:** hypertrophic cardiomyopathy, obstructive hypertrophic cardiomyopathy, nonobstructive hypertrophic cardiomyopathy, electrocardiogram, classification

## Abstract

**Background:**

The clinical presentation and prognosis of hypertrophic cardiomyopathy (HCM) are heterogeneous between nonobstructive HCM (HNCM) and obstructive HCM (HOCM). Electrocardiography (ECG) has been used as a screening tool for HCM. However, it is still unclear whether the features presented on ECG could be used for the initial classification of HOCM and HNCM.

**Objective:**

We aimed to develop a pragmatic model based on common 12-lead ECG features for the initial identification of HOCM/HNCM.

**Methods:**

Between April 1^st^ and September 30^th^, 2020, 172 consecutive HCM patients from the International Cooperation Center for Hypertrophic Cardiomyopathy of Xijing Hospital were prospectively included in the training cohort. Between January 4^th^ and February 30^th^, 2021, an additional 62 HCM patients were prospectively included in the temporal internal validation cohort. External validation was performed using retrospectively collected ECG data with definite classification (390 HOCM and 499 HNCM ECG samples) from January 1^st^, 2010 to March 31^st^, 2020. Multivariable backward logistic regression (LR) was used to develop the prediction model. The discrimination performance, calibration and clinical utility of the model were evaluated.

**Results:**

Of all 30 acquired ECG parameters, 10 variables were significantly different between HOCM and HNCM (all *P* < 0.05). The P wave interval and SV1 were selected to construct the model, which had a clearly useful C-statistic of 0.805 (0.697, 0.914) in the temporal validation cohort and 0.776 (0.746, 0.806) in the external validation cohort for differentiating HOCM from HNCM. The calibration plot, decision curve analysis, and clinical impact curve indicated that the model had good fitness and clinical utility.

**Conclusion:**

The pragmatic model constructed by the P wave interval and SV1 had a clearly useful ability to discriminate HOCM from HNCM. The model might potentially serve as an initial classification of HCM before referring patients to dedicated centers and specialists.

**Highlights:**

**What are the novel findings of this work?**

- Evident differences exist in the ECG presentations between HOCM and HNCM.
- To the best of our knowledge, this study is the first piece of evidence to quantify the difference in the ECG presentations between HOCM and HNCM.
- Based on routine 12-lead ECG data, a probabilistic model was generated that might assist in the initial classification of HCM patients.

## Introduction

Hypertrophic cardiomyopathy (HCM) is one of the most common genetic cardiovascular diseases, with a large heterogeneity in its clinical presentation and prognosis. Obstructive hypertrophic cardiomyopathy (HOCM) accounts for two-thirds of HCM cases. It is a state in which the myocardium is highly contracted, causing left ventricular outflow tract obstruction (LVOTO) and leading to severe symptoms and adverse events [1,2].

The prognosis and management of HOCM and nonobstructive HCM (HNCM) are different. Patients with HOCM usually experience a high overall risk of advanced heart failure (HF) and atrial fibrillation (AF) [1,3], whereas the majority of HNCM patients are usually asymptomatic or have only mild symptoms. It has been considered that HNCM patients are associated with a low probability of HF or other major adverse consequences and do not require surgical interventions [4]. Septal reduction therapy (SRT) was recommended for symptomatic HOCM patients who progressed to drug refractory HF [5]. It has been suggested that referring HOCM patients to high-volume and high-expertise centers receiving SRT might lead to good outcomes with lower procedural mortality, lower costs and bleeding complications, and improvement in clinical discomforts [6,7]. Thus, early classification of HNCM and HOCM is important for medical counseling, prompt referral, and even longitudinal clinical follow-up.

The diagnosis of HCM and the subsequent categorization of HOCM and HNCM relies mostly on the measurement of the maximum wall thickness (MWT) and left ventricular outflow tract gradient (LVOTG≥30 mmHg) by echocardiography (Echo) or stress Echo [8,9]. However, instant, routine Echo in patients suspected to have HCM is less practical in rural or undeveloped regions due to the high cost, lack of infrastructure or well-trained cardiac sonographic specialists [10]. Most HCM patients have electrocardiographic abnormalities [11], and 12-lead electrocardiography (ECG) might offer an attractive noninvasive, low-cost, and convenient approach to screen for HCM. Generally, ECG screening has relied on particular features, such as left axis deviation, left ventricular high voltage, prominent Q waves, and T-wave inversions. Nevertheless, these single features have a less satisfied diagnostic performance [12,13]. Prediction models based on ECG using traditional statistical methods [14] or artificial intelligence [15,16] have achieved high accuracy in diagnosing or risk stratifying HCM. However, it is still unclear whether the features presented on ECG could be used for the initial classification of HNCM and HOCM.

In the current study, we constructed a practicable model based on the most common and easily available ECG features to distinguish HOCM from HNCM, with the aim to assist in the initial classification of HCM patients.

## Methods

### Study population

**Figure 1:**
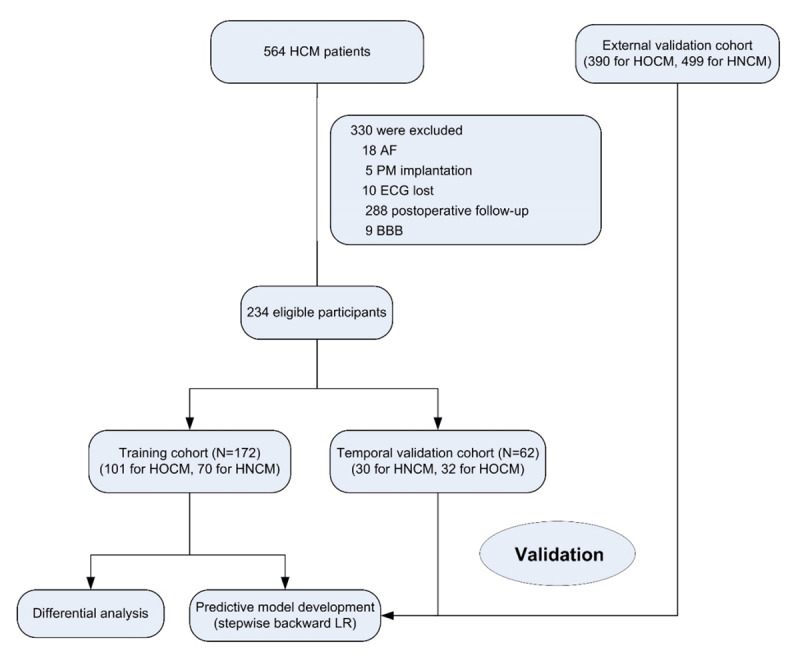
Study flow chart. AF, atrial fibrillation; PM, pacemaker; BBB, bundle branch block; HCM, hypertrophic cardiomyopathy; HOCM, obstructive hypertrophic cardiomyopathy; HNCM, non-obstructive hypertrophic cardiomyopathy; LR, logistic regression.

Between April 1^st^ and September 30^th^, 2020, 172 consecutive HCM patients—102 HOCM and 72 HNCM patients—from the International Cooperation Center for Hypertrophic Cardiomyopathy of Xijing Hospital were prospectively included in the training cohort. Between January 4^th^ and February 30^th^, 2021, an additional 62 HCM patients from the same center were prospectively included in the temporal internal validation cohort (30 HOCM and 32 HNCM patients). External validation was performed using retrospectively collected ECG data with definite classification of HOCM or HNCM (390 HOCM and 499 HNCM ECG samples) from Xijing Hospital by the end of March 2020. The study flowchart is shown in Figure 1. Patients who had previously received an interventricular reduction procedure, had a pacemaker with pacing rhythm, had persistent atrial fibrillation (AF), had bundle branch block (BBB), or had missing ECG data were excluded. The enrolled HCM participants predominantly came from Northwest, Central, Northern, and Eastern China (Supplemental Figure S1).

The research protocol was approved by the ethics committee of Xijing Hospital, and the requirement for written informed consent was waived by the institutional review board. The study was performed in accordance with the local laws and the regulations of Xijing Hospital, and the study complied with the Declaration of Helsinki.

### Electrocardiogram Evaluation

All participants underwent routine 12-lead ECG in the supine position at a sampling rate of 500 Hz, an amplitude of 10 mm/mv and a speed of 25 mm/s. The ECG features were measured automatically by the computer, and these data were checked independently by two experienced ECG reviewers blinded to the clinical details [14].

A total of 30 common ECG parameters were acquired, including the mean heart rate (HR), P wave duration, intervals of PR, QT and the corrected QT (QTc), abnormal Q wave, T wave inversion (TWI), and the amplitudes of the R and S waves in all precordial leads (V1–V6) and in limb leads I, III, and aVL (expressed as “R/S+Lead”). The amplitudes of RV5 + SV1 (RV5SV1, Sokolov–Lyon index), RaVL + SV3 (RaVLSV3, Cornell index), and RI + SIII (RISIII) were calculated for reflecting pathological high LV wall voltage, and the amplitudes of the R and S waves in leads V1–4, reflecting interventricular septum (IVS) hypertrophy, were also included.

### Echocardiography acquisition

All subjects underwent a transthoracic two-dimensional and Doppler Echo that was independently performed by two experienced cardiac sonographers. A maximum LVOTG (LVOTG_max_) of ≥ 30 mmHg is classified as HOCM; conversely, a rest and stress LVOTG_max_ < 30 mmHg is HNCM [17,18].

All the echocardiographic and Doppler measurements were averaged from three cardiac cycles, and the measurements included the biatrial dimension, left ventricular end-systolic volume (ESV), left ventricular end-diastolic volume (EDV), left ventricular ejection fraction (LVEF), left ventricular outflow tract pressure gradient (LVOTG), and maximum left ventricular wall thickness (MWT). Tissue Doppler signals were collected from the mitral inflow and mitral annulus tissue, the early (E) and late (A) diastolic mitral inflow velocity; the early (E’) and late (A’) diastolic mitral annular tissue velocity were recorded at 100 mm/s, respectively [19]. E/A and E/E’ were calculated to reflect the left ventricular diastolic function. The SAM sign, that is the forward motion of the mitral valve during systole, is an abnormal waveform of the mitral valve leaflet removed to the ventricular septum during systole. All the above procedures were in accordance with the guidelines of the American Society of Echocardiography and the European Society for the Quantification of Cardiac Chambers in Adult Echocardiography [20].

### Statistical analysis

The sample size was derived based on an attempt to include all available samples during the study interval, and no power calculation was performed in advance. Categorical variables were expressed as the frequency and percentage. Normally distributed continuous variables were expressed as the mean with standard deviations (SD) or median [25^th^ and 75^th^ percentiles], depending on their distribution. Between groups comparisons, including t test and nonparametric Mann-Whitney U tests, were performed as appropriate for continuous variables, and Fisher’s exact tests were used for categorical variables.

Univariable and multivariable logistic regression (LR) analyses with backward stepwise-regression were used to screen variables and construct the model. The discrimination accuracy was quantified by receiver operating characteristic (ROC) curves using the C-statistic. According to previous literature [21], a C-statistic greater than 0.75 reflects a clearly useful discrimination; C-statistic less than 0.60 reflects poor discrimination; 0.60 to 0.75, possibly helpful discrimination. A calibration curve with the R package ‘CalibrationCurves’ was used to assess the goodness-of-fit of the model. Decision curve analysis (DCA) and clinical impact curve (CIC) analysis with the R package ‘rmda’ was conducted to assess the clinical effectiveness of the model. For any given patient’s probability threshold, the DCA curve with the highest benefit score at that threshold is the best choice [22]. An online browser-based calculator (http://121.36.159.143:9999/hocm.do) was generated accordingly.

All statistical analyses were carried out using R 3.6.1 software and SPSS 26.0; a two-tailed *P* value < 0.05 was considered to be statistically significant.

## Results

### Baseline characteristics

Of the total 234 prospectively enrolled participants, 172 were included in the training cohort and the remaining 62 participants were included in the temporal validation cohort. The baseline characteristics were presented in Table 1. The median ages were 47 and 46 years in HOCM and HNCM group, respectively, and males accounted for more than 60% of the patients in both groups. There was no significant difference in the diameters of right atrium (RA), end-diastolic volume (EDV), end-systolic volume (ESV), left-ventricular ejection fraction (LVEF), and E/A between the HOCM and HNCM groups (all *P* > 0.05). The HOCM group had larger left atrium dimension (LAD), maximal wall thickness (MWT), LVOTG, E/E’ and more positive systolic anterior motion (SAM) sign (all *P* < 0.05).

**Table 1:** Baseline characteristics.


	ALL			TRAINING COHORT			TEMPORAL VALIDATION COHORT		
		
	HOCM N = 132	HNCM N = 102	*P*	HOCM N = 102	HNCM N = 70	*P*	HOCM N = 30	HNCM N = 32	*P*

Age, y (mean, SD)	47 (14)	46 (15)	0.635	47 (14)	47 (15)	0.962	48 (15)	44 (15)	0.372

Male (n, %)	97 (73.48)	66 (64.71)	0.147	74 (72.55)	46 (65.71)	0.338	23 (76.67)	20 (62.50)	0.227

**Echocardiography parameters**									

MWT, mm	23 [20, 27]	20 [17, 23]	<0.001	23 [20, 27]	19 [17, 24]	<0.001	24 [20, 27]	20 [16, 22]	0.003

RAD, mm	34 [32, 36]	35 [32, 37]	0.092	34 [31, 36]	35 [31, 37]	0.091	35 [33, 36]	34 [32, 37]	0.854

LAD, mm	44 [41, 48]	40 [37, 45]	<0.001	44 [40, 48]	41 [37, 45]	<0.001	45 [42, 48]	40 [37, 45]	0.001

EDV, ml	78 [65, 88]	77 [67, 90]	0.532	78 [65, 87]	80 [69, 91]	0.174	80 [71, 91]	74 [62, 89]	0.269

ESV, ml	31 [25, 37]	32 [27, 39]	0.186	31 [25, 36]	33 [27, 39]	0.126	32 [26, 38]	31 [27, 39]	0.972

LVEF, %	59 [57, 62]	58 [56, 61]	0.090	59 [57, 62]	58 [56, 61]	0.137	59 [57, 62]	59 [57, 61]	0.548

E/E’	14.48 [11.15, 20.48]	11.13 [9.31, 13.87]	<0.001	15.17 [11.69, 20.90]	11.56 [9.31, 13.87]	<0.001	13.40 [10.92, 16.43]	10.95 [9.63, 14.05]	0.046

E/A	0.81 [0.66, 1.32]	0.84 [0.73, 1.35]	0.296	0.80 [0.66, 1.28]	0.85 [0.75, 1.35]	0.136	0.85 [0.66, 1.44]	0.77 [0.65, 1.35]	0.667

LVOTGmax, mmHg	58 [22, 96]	5 [3, 7]	<0.001	64 [22, 99]	5 [4, 7]	<0.001	71 [35, 102]	6 [4, 10]	<0.001

SAM sign [n, %]	112 (84.85)	10 (9.80)	<0.001	87 (85.29)	9 (12.86)	<0.001	25 (83.33)	1 (3.13)	<0.001


Data are expressed as n (%), mean (SD), or median [25^th^ and 75^th^ percentiles], otherwise specified. MWT, maximum wall thickness; RA, right atrium dimension; LA, left atrium dimension; EDV, end-diastolic volume; ESV, end-systolic volume; LVEF, left ventricular ejection fraction; E, early diastolic mitral inflow velocity; A, late diastolic mitral inflow velocity; E’, early diastolic mitral annular tissue velocity; A’, late diastolic mitral annular tissue velocity; E/A, ratio of peak velocities of the early diastolic peak and late peak in mitral inflow; E/E’, ratio of early diastolic peak velocity in mitral inflow to mitral annular; LVOTGmax, the maximum of left ventricular outflow tract gradient; SAM, systolic anterior motion.

### ECG parameters

Among the total 30 obtained ECG variables, compared with HNCM, HOCM was associated with significantly larger P wave interval and higher amplitudes in SV1, RI, RaVL, SV2, SV5, SV6, RV5SV1, RISIII, and SV1V2 (all *P* < 0.05). There was no significant difference in the other 20 ECG variables (Table 2).

**Table 2:** ECG parameters.


	ALL			TRAINING COHORT			TEMPORAL VALIDATION COHORT		
		
	HOCM N = 132	HNCM N = 102	*P*	HOCM N = 102	HNCM N = 70	*P*	HOCM N = 30	HNCM N = 32	*P*

P, ms	106 [100, 114]	100 [92, 107]	<0.001	104 [100, 110]	100 [90, 106]	<0.001	112 [104, 122]	100 [93, 108]	<0.001

QRS, ms	104 [94, 112]	100 [92, 111]	0.408	104 [92, 112]	104 [92, 114]	0.951	104 [96, 107]	96 [92, 108]	0.094

PR, ms	154 [139, 172]	148 [136, 164]	0.117	151 [136, 172]	152 [138, 164]	0.720	160 [146, 176]	146 [133, 163]	0.009

QTc, ms	430 [411, 445]	426 [413, 441]	0.441	428 [410, 442]	427[414, 441]	0.965	433 [416, 454]	422 [410, 443]	0.102

Abnormal Q	37 (28.03)	19 (18.63)	0.095	26 (25.49)	10 (14.29)	0.088	11 (36.67)	9 (28.13)	0.589

TWI, %	80 (60.61)	61 (59.80)	0.901	62 (60.78)	41 (58.57)	0.874	18 (60.00)	20 (62.50)	>0.999

RI, mv	1.20 [0.80, 1.68]	0.80 [0.50, 1.23]	<0.001	1.29 [0.80, 1.70]	0.90 [0.50, 1.20]	0.001	1.05 [0.70, 1.40]	0.80 [0.60, 1.38]	0.133

RV1, mv	0.31 [0.10, 0.70]	0.40 [0.20, 0.80]	0.535	0.31 [0.10, 0.70]	0.40 [0.20, 0.80]	0.267	0.35 [0.10, 0.70]	0.55 [0.26, 1.20]	0.055

RV2, mv	0.80 [0.30, 1.50]	1.03 [0.52, 1.90]	0.047	0.80 [0.30, 1.50]	0.80 [0.50, 1.55]	0.450	0.74 [0.29, 1.76]	1.35 [0.81, 2.30]	0.036

RV3, mv	1.75 [0.90, 2.60]	1.45 [0.95, 2.50]	0.523	1.65 [0.79, 2.60]	1.35 [0.80, 2.50]	0.457	2.23 [1.07, 2.70]	1.95 [1.10, 2.40]	0.587

RV4, mv	2.60 [1.74, 3.68]	2.35 [1.39, 3.50]	0.154	2.60 [1.72, 3.70]	2.45 [1.38, 3.33]	0.294	2.70 [1.93, 3.50]	1.95 [1.39, 3.78]	0.310

RV5, mv	2.70 [1.70, 3.55]	2.35 [1.30, 3.36]	0.072	2.61 [1.60, 3.70]	2.60 [1.30, 3.35]	0.210	2.85 [1.95, 3.21]	1.71 [1.29, 3.39]	0.178

RV6, mv	2.03 [1.40, 2.80]	1.80 [1.10, 2.70]	0.070	2.00 [1.30, 3.00]	1.85 [1.08, 2.63]	0.159	2.30 [1.64, 2.80]	1.68 [1.16, 2.78]	0.215

RaVL, mv	0.60 [0.38, 1.12]	0.50 [0.20, 1.23]	0.019	0.64 [0.40, 1.13]	0.50 [0.20, 1.00]	0.032	0.45 [0.33, 1.12]	0.50 [0.23, 0.78]	0.521

SIII, mv	0.30 [0.00, 0.79]	0.30 [0.00, 0.70]	0.595	0.30 [0.00, 0.72]	0.30 [0.00, 0.70]	0.685	0.41 [0.00, 0.85]	0.30 [0.00, 0.72]	0.622

SV1, mv	2.10 [1.40, 2.76]	1.30 [0.92, 1.73]	<0.001	2.10 [1.40, 2.80]	1.30 [0.90, 1.80]	<0.001	2.00 [1.25, 2.72]	1.39 [1.03, 1.69]	0.007

SV2, mv	2.30 [1.50, 3.17]	1.90 [1.30, 2.50]	0.008	2.30 [1.50, 3.11]	1.85 [1.20, 2.50]	0.029	2.35 [1.54, 3.83]	2.00 [1.40, 2.48]	0.128

SV3, mv	1.50 [0.68, 2.38]	1.50 [0.80, 2.40]	0.634	1.50 [0.70, 2.30]	1.50 [0.78, 2.40]	0.576	1.73 [0.44, 2.50]	1.49 [0.93, 2.38]	0.966

SV4, mv	0.90 [0.40, 1.78]	1.03 [0.58, 1.90]	0.135	0.90 [0.38, 1.53]	1.20 [0.58, 1.90]	0.084	0.85 [0.43, 2.03]	0.95 [0.53, 1.64]	0.933

SV5, mv	0.40 [0.10, 0.90]	0.68 [0.30, 1.10]	0.005	0.35 [0.00, 0.90]	0.70 [0.30, 1.13]	0.004	0.42 [0.20, 1.26]	0.50 [0.30, 1.08]	0.708

SV6, mv	0.20 [0.00, 0.40]	0.30 [0.00, 0.60]	0.018	0.20 [0.00, 0.30]	0.25 [0.00, 0.60]	0.030	0.27 [0.08, 0.53]	0.30 [0.15, 0.59]	0.645

RV5SV1, mv	4.58 [3.44, 6.16]	3.50 [2.50, 4.91]	<0.001	4.60 [3.20, 6.30]	3.55 [2.40, 4.95]	0.001	4.58 [3.90, 5.81]	3.35 [2.50, 4.93]	0.010

RⅠSⅢ, mv	1.50 [1.00, 2.28]	1.25 [0.70, 1.93]	0.005	1.53 [1.00, 2.25]	1.25 [0.68, 2.00]	0.014	1.40 [1.00, 2.34]	1.20 [0.80, 1.90]	0.231

RaVLSV3, mv	2.30 [1.38, 3.19]	2.30 [1.30, 2.86]	0.598	2.30 [1.40, 3.06]	2.30 [1.22, 2.90]	0.711	2.23 [1.13, 3.79]	2.20 [1.30, 2.80]	0.688

RV2V3, mv	2.60 [1.40, 4.22]	2.55 [1.50, 4.23]	0.573	2.53 [1.40, 4.19]	2.30 [1.40, 4.07]	0.821	3.00 [1.41, 4.73]	2.95 [2.23, 5.05]	0.375

SV2V3, mv	3.95 [2.21, 5.16]	3.50 [2.28, 4.80]	0.186	3.85 [2.22, 4.90]	3.55 [2.25, 4.80]	0.377	4.68 [1.99, 5.63]	3.48 [2.25, 4.80]	0.254

RV1V2, mv	1.25 [0.44, 2.15]	1.43 [0.90, 2.62]	0.042	1.30 [0.43, 2.12]	1.30 [0.70, 2.33]	0.388	1.08 [0.43, 2.53]	2.03 [1.13, 3.51]	0.029

SV1V2, mv	4.46 [3.13, 5.70]	3.20 [2.37, 4.13]	<0.001	4.37 [2.98, 5.83]	3.30 [2.00, 4.45]	<0.001	4.48 [3.30, 5.48]	3.20 [2.41, 3.99]	0.005

RV3V4, mv	4.35 [2.62, 6.08]	3.90 [2.30, 6.00]	0.292	4.15 [2.58, 6.10]	3.95 [2.18, 5.93]	0.382	5.15 [3.11, 5.93]	3.85 [2.50, 6.25]	0.517

SV3SV4, mv	2.50 [0.99, 4.21]	2.60 [1.39, 4.30]	0.358	2.45 [1.06, 3.92]	2.80 [1.30, 4.30]	0.235	2.58 [0.82, 4.81]	2.40 [1.45, 4.09]	0.816


Data are expressed as n (%) or median [25^th^ and 75^th^ percentiles]. TWI, T wave inversion; RV5SV1, amplitude of the R wave in V5 plus the S wave amplitude in V1; RⅠSⅢ, amplitude of the R wave in lead Ⅰ plus the S wave amplitude in lead Ⅲ; RaVLSV3, amplitude of the R wave in lead aVL plus the S wave amplitude in precordial lead V3; RV2V3, amplitude of the R wave in precordial lead V2 plus V3; SV2V3, amplitude of the S wave in precordial lead V2 plus V3. The rest set as the analogy.

**Selection of the predictors and construction of the HOCM model**

All the above 10 ECG variables with statistical significance were incorporated into the univariable and multivariable LR with backward stepwise selection (enter 0.1, removal 0.01). Two variables, P wave interval (P) and the amplitude of the S wave in lead V1 (SV1), were found to be independent predictors of HOCM (Table 3). The formula of the model is presented as follows:

**Table 3:** Univariable and multivariable logistic regression analyses.


VARIABLES	UNIVARIABLE ANALYSIS			MULTIVARIABLE ANALYSIS		
	
	COEFFICIENT	OR (95% CI)	*P*	COEFFICIENT	OR (95% CI)	P

RaVL	0.36	1.43 (0.88, 2.31)	0.150			

SV1V2	0.34	1.40 (1.17, 1.68)	<0.001			

RⅠSⅢ	0.25	1.29 (0.97, 1.71)	0.081			

SV6	–0.48	0.62 (0.34, 1.13)	0.116			

RⅠ	0.73	2.07 (1.26, 3.41)	<0.001			

RV5SV1	0.31	1.37 (1.14, 1.65)	0.001			

SV2	0.35	1.42 (1.06, 1.90)	0.017			

SV5	–0.47	0.63 (0.41, 0.96)	0.032			

P	0.07	1.07 (1.04, 1.11)	<0.001	0.068	1.07 (1.04, 1.11)	<0.001

SV1	0.91	2.49 (1.67, 3.72)	<0.001	0.930	2.54 (1.65, 3.89)	<0.001

Constant				–8.135		



{\rm{Y}}\ =\ - 8.135\ +\ 0.068*{\rm{P}}\ +\ 0.930*{\rm{SV1}}

### The calibration and discrimination performance of the model

**Figure 2:**
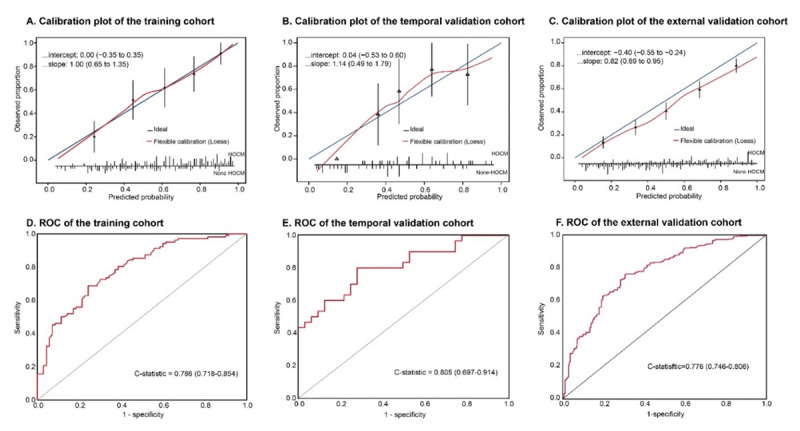
The calibration and ROC curve of the model in the training, temporal, and external validation cohorts. Calibration plots between the predicted and observed HOCM patients in the training **(A)**, temporal **(B)**, and external validation **(C)** cohorts. The 45° blue line represents a perfect prediction, and the red line represents the predictive performance of the model. ROC curves of the training **(D)**, temporal **(E)**, and external **(F)** validation cohorts.

Calibration curves were generated to evaluate the fitness of the model. The calibration plots presented slopes of 1.00 (0.65~1.35), 1.14 (0.49, 1.79) and 0.82 (0.69, 0.95), intercepts of 0.00 (–0.35~0.35), 0.04 (–0.53, 0.60) and –0.40 (–0.55, –0.24) in the training, temporal, and external validation cohorts (Figure 2A–C), respectively. The model had a clearly useful C-statistic of 0.786 (0.718–0.854) in the training cohort, 0.805 (0.697–0.914) in the temporal validation cohort, and 0.776 (0.746–0.806) in the external validation cohort for differentiating HOCM from HNCM (Figure 2D–F). When the cutoff was set at 0.53 based on the Youden index, the sensitivity, specificity, and accuracy were 0.80, 0.72, and 0.71, respectively.

### The correlation between LVOTG and the prediction score value

**Figure 3:**
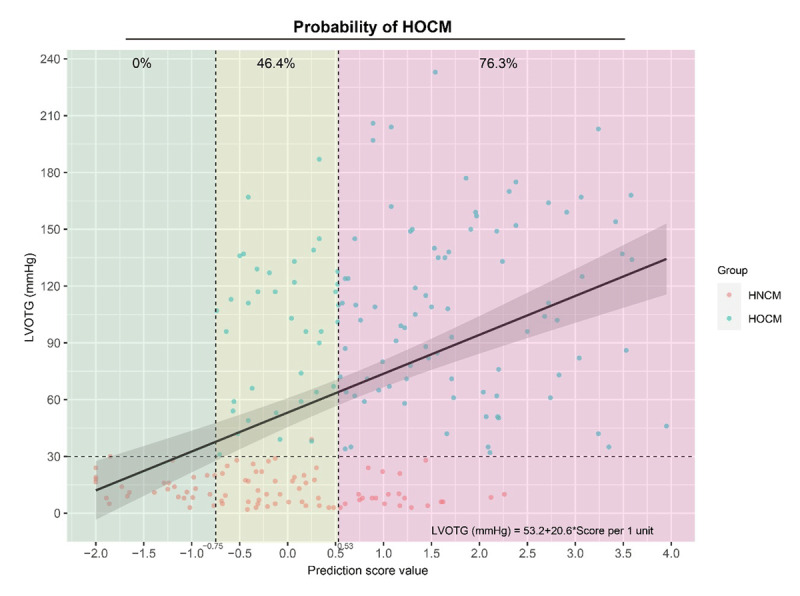
The relationship between LVOTG and the prediction score value.

A scatter plot was used to illustrate the association between LVOTG (stress echo) and the prediction score value (Figure 3). We found that LVOTG had a linear relationship (P < 0.001) with the prediction score value (LVOTG = 53.2 + 20.6 * score per 1 unit). The risk stratification strata are presented in Figure 3. In the ‘green zone’ (the prediction score value < –0.75), the probability of HOCM was 0%, whereas in the ‘yellow zone’ (the prediction score value ranged from –0.75 to 0.53) and ‘red zone’ (the prediction score value > 0.53), the probability of HOCM was 46.4% and 76.3%, respectively.

### The utility of the model for assessing HOCM probability

Decision curve analysis (DCA) was used to estimate the net benefits of the developed model at different thresholds, with the probability thresholds on the horizontal axis and the net benefit scores on the vertical axis. The model could gain more net benefits than either ‘none’ or ‘all’ HOCM patients recognized when the threshold probability was between 20% and 90%, which indicates a high cost-efficient net and potential for clinical application (Supplemental Figure 2A). Furthermore, clinical impact curve (CIC) was utilized to demonstrate the clinical effectiveness of the model and to predict the probability of HOCM among 1000 samples. When the threshold probability was greater than 80%, the predictive number of HOCM was approximately the same as the actual number (Supplemental Figure 2B).

### Examples illustration

**Figure 4:**
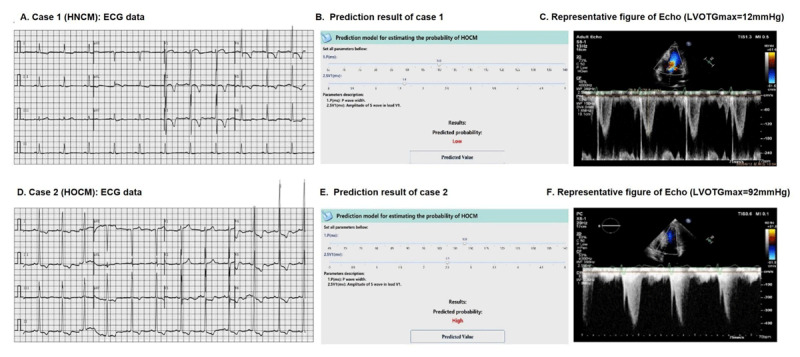
Examples illustration. A, The ECG of the patient from case 1 (HNCM); B, the prediction result of case 1 with a low HOCM probability; C, Peak LVOTG (12 mmHg) of the patient from case 1; D, The ECG of the patient from case 2 (HOCM); E, the prediction result of case 2 with a high HOCM probability; F, Peak LVOTG (92 mmHg) of the patient from case 2.

Two case scenarios are shown in Figure 4. Case 1 was a 58-year-old male patient with chest discomfort during activity. ECG showed that the patient had a sinus rhythm with a P wave interval of 96 ms, SV1 of 1.6 mv, and TWI in precordial leads of V2–V5 (Figure 4A). Our previous calculator, which could be used to screen for HCM/non-HCM, (http://121.36.159.143:9999/hcm.do) indicated that the patient had a high probability of HCM, and the present model further indicated a low probability of HOCM (Figure 4B). After examination by Echo, the patient was diagnosed as HNCM with a MWT of 21 mm and LVOTG_max_ of 12 mmHg (Figure 4C). Finally, the patient was prescribed β-blockers. Case 2 was a 40-year-old male with a family history of HCM. ECG showed the patient had sinus rhythm with a P wave interval of 108 ms, SV1 of 2.5 mv, and TWI in the inferior and precordial leads (Figure 4D). The prediction model suggested a high HCM probability with LVOT obstruction (Figure 4E). The patient was referred to our HCM center, and the Echo showed a MWT of 26 mm and a LVOTGmax of 92 mmHg (Figure 4F). The patient was diagnosed with HOCM and received the SRT procedure by percutaneous intramyocardial septal radiofrequency ablation [23].

## Discussion

In this study, we found that there are differences in the ECG presentations between HOCM and HNCM. To the best of our knowledge, this study is the first piece of evidence to quantify the difference in ECG presentations between HOCM and HNCM, and a prediction model was constructed to categorize these two subtypes of HCM.

Surface 12-leads ECG is a ubiquitous and less resource-intensive approach in clinical practice. Studies have suggested that prediction models based on ECG can achieve high accuracy in detecting HCM and have proposed that patients who are suspected of having HCM should undergo routine ECG, which could reflect the morphology and function of the heart [24].

The HOCM and HNCM are the two subtypes of HCM and are associated with distinct prognosis and treatment strategies. A previous study [25] showed that there were more abnormal ECG presentations in HOCM than in HNCM; the ECG parameters reflected left ventricular hypertrophy (RV5, RV6, SV1, SV2, ST-T change, etc.), and left atrial abnormalities were also more commonly presented in HOCM than in HNCM. Recently, researchers reported an ECG model using artificial intelligence for the assessment of disease status and treatment response in HOCM [26]. However, it is still debatable whether an abnormal ECG could to some extent reflect LVOTG assessed by Echo. In addition, it is still unclear whether the features presented on ECG could be used for the initial classification of HOCM and HNCM. In this study, a feasible model consisting of the P wave interval and SV1 showed clearly useful discrimination of HOCM from HNCM, and we found that the prediction risk score was associated with LVOTG, which might be used to reflect LVOTG.

In the current formula, SV1 was included to predict HOCM/HNCM. The increase of SV1, the R-wave amplitude in the leads facing the left ventricle (I, aVL, V5 and V6), and the deepening of the S-wave amplitude in the leads V2/V3 were considered as ECG markers reflecting left ventricular hypertrophy (LVH) [27,28]. However, it has been suggested that these left ventricular hypertrophy markers alone were not associated with a higher LVOTG, and that these markers had poor discrimination ability for differentiating HOCM from HNCM [25].

Similarly, it has also been reported that the AUC of the ROC curve for left ventricular hypertrophy by the ECG voltage criteria alone was only 0.675 [29]. It suggested a ‘possibly helpful’ discrimination ability (AUC: 0.60–0.75) for severe aortic stenosis (AS) detection according to the guides of discrimination and calibration of clinical prediction models [21]. In our study, the amplitudes of the ECG markers traditionally used for indicating left ventricular hypertrophy (RI, RaVL, SV1, SV2, RV5SV1, RISIII, and SV1V2) were also all higher in HOCM than in HNCM. However, we found that SV1 was the strongest predictor among these variables in the logistic regression model.

The P wave interval was also incorporated into the prediction model. The P wave interval has been recognized to be correlated with the left atrial volume, and a prolonged P wave interval is associated with delayed interatrial conduction [30,31,32,33]. Compared to HNCM, the prolonged P wave interval showed in the HOCM group could be explained by the fact that HOCM patients typically have more severe impaired diastolic function and atrial dysfunction [34,35]. Concordantly, we observed that left atrial dimension assessed by Echo was also larger in HOCM than in HNCM.

The diagnosis of HOCM relies on the measurement of LVOTG by Echo. However, the LVOTG was not measured routinely, and it might not be accurately measured by less well-trained medical staff. Furthermore, the variability of LVOTG was reported to be as high as 49.0 mmHg in the absence of provocative maneuvers or interventions, which may result in discrepant classification [36,37]. A 12-lead ECG could offer a noninvasive, low-cost, and rapid means of screening for cardiovascular diseases. It has been suggested that the increase of SV1 was significantly reduced by 90% after HOCM patients who received intramyocardial radiofrequency ablation procedure to alleviate LVOTG [38]. Similarly, based on the 12-lead ECG, a deep learning-based algorithm was verified with high accuracy for severe AS (mean gradient pressure 32 mmHg) screening [39]. Another study also reported that AI-based ECG could mirror the decreasing trends over time in LVOTG (>100 mmHg pretreatment to less than 30 mmHg until the end of the study) for HOCM patients receiving mavacamten [26]. Such evidence indicates that ECG abnormalities may be associated with LVOTG. However, the precise features that the AI sees are obscure, AI needs advanced infrastructures, and the model may not be accessible for everyone, especially in undeveloped regions or communities.

In the current study, in virtue of the classical statistical approach, we found that the LVOTG had a linear relationship with the score of the prediction model, and the model may be regarded as a potential tool to ‘translate’ ECG features to LVOTG without dedicated instrument.

### Limitations

First, the participants enrolled in the current study were from a single center and were all Chinese. Further external validation using participants from multiple centers and a population with more heterogeneity is needed. Second, the variables included in the formula of the model focused only on those parameters that are commonly assessed in clinical practice to increase the simplicity and applicability of the model; thus, some important but less frequently used features might have been omitted. Third, although some researchers reported that ECG was correlated with the HCM phenotype rather than the genotype [40], the effects of the genotype of HCM patients was not taken into consideration.

## Conclusion

There are differences in the ECG presentations between HOCM and HNCM. The pragmatic model constructed by the commonly used parameters of P wave interval and SV1 had ‘clearly useful’ discrimination of the HCM subtypes. Such a model might assist the initial classification of suspected HCM patients and has potential in the follow-up of disease progression or longitudinal monitoring of treatment response.

## Additional File

The additional file for this article can be found as follows:

10.5334/gh.1250.s1 Supplemental Figures. Figures 1 and 2.

## Figures and Tables

**Table:** 

AF	Atrial fibrillation
BBB	Bundle branch block
CI	Confidence intervals
CIC	Clinical impact curve
DCA	Decision curve analysis
ECG	Electrocardiogram
Echo	Echocardiography
EDV	End-diastolic volume
ESV	End-systolic volume
HCM	Hypertrophic cardiomyopathy
HNCM	Nonobstructive HCM
HOCM	Obstructive HCM
LAD	Left atrium dimension
LR	Logistic regression
LV	Left ventricular
LVEF	Left ventricular ejection fraction
LVH	Left ventricular hypertrophy
LVOTG	Left ventricular outflow tract gradient
LVOTO	Left ventricular outflow obstruction
MWT	Maximum wall thickness
RA	Right atrium
ROC	Receiver operator characteristic
RV5SV1	The sum amplitude of R wave in lead V5 And S wave in lead V1
SAM	Systolic anterior motion
SRT	Septal reduction therapy
SV1	The amplitude of S Wave in Lead V1
