# Supplementary material for: Identifying Obstructive Hypertrophic Cardiomyopathy from Nonobstructive Hypertrophic Cardiomyopathy: Development and Validation of a Model Based on Electrocardiogram Features
Source: Glob Heart. 2023 Aug 4;18(1):40. doi: 10.5334/gh.1250 (PMC10402817; doi:10.5334/gh.1250)
Supplement: Supplemental Figures. — Figures 1 and 2. [file gh-18-1-1250-s1.pdf]

Supplemental figures and figure legends

Supplemental Figure 1. Distribution of all the enrolled HCM participants

Supplemental Figure 2. The results of DCA and CIC of the model for assessing HOCM in the training cohort

DCA for the model in the training cohort (A). DCA presented as a curve with the probability thresholds on the horizontal axis and the benefit score on the vertical axis. The black line indicates the net benefit of none identification of HOCM, and the gray line indicates the net benefit of all identified HOCM patients. The blue line denotes the net benefit curve at different predictive probabilities. The model could gain more net benefits than the all-or-none patients recognized, which indicates a high potential for clinical applications. CIC is shown in figure B to predict the number of HOCM patients within a population size of 1000. The blue solid curve denotes the predicted HOCM number of patients at different threshold probabilities, and the dotted curve shows the actual patient number. The predictive number of HOCM patients was approximate to the actual number when the threshold probability was above 0.8.

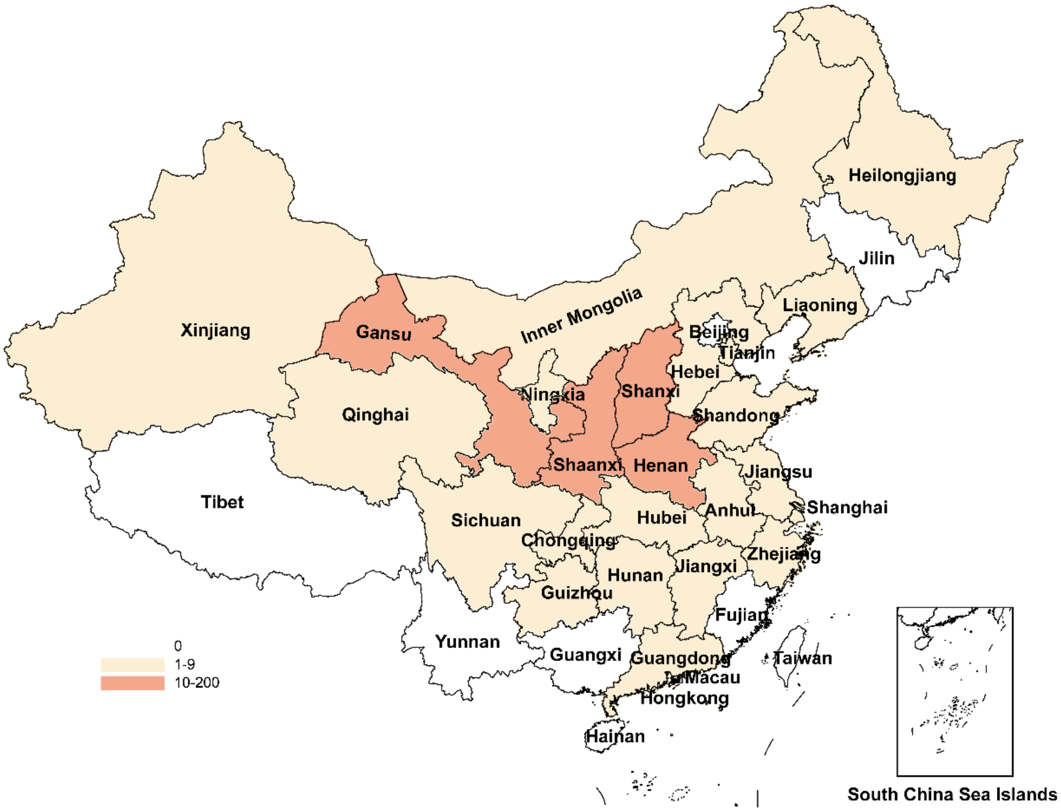

Supplemental Figure 1. Distribution of all the enrolled HCM participants

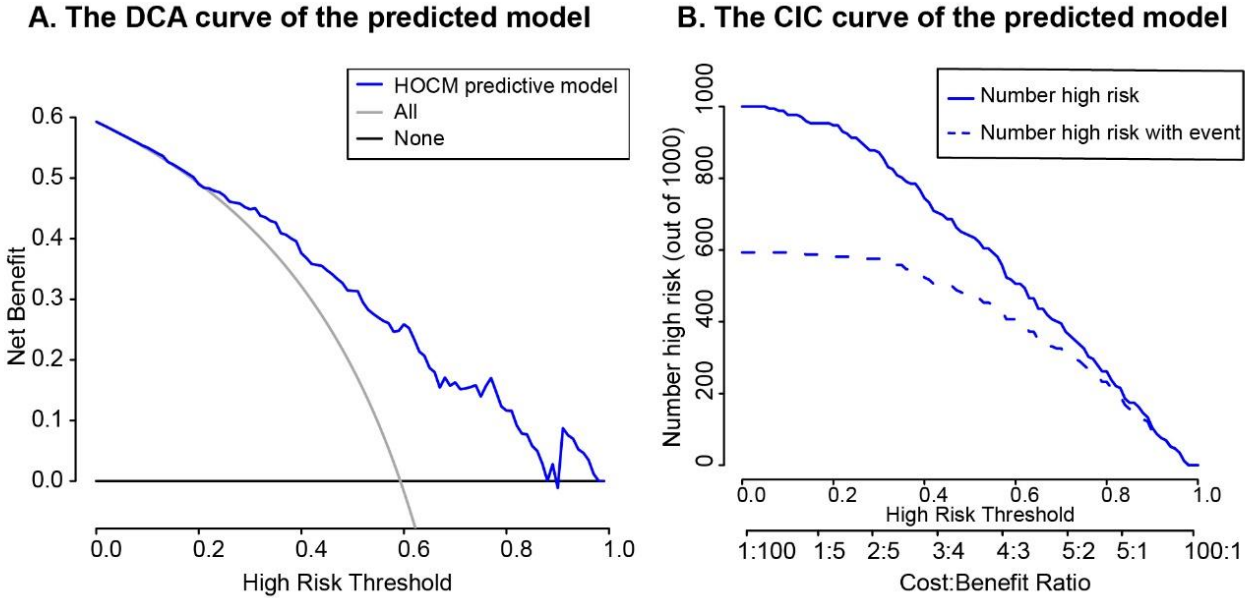

Supplemental Figure 2. The results of DCA and CIC of the model for predicting HOCM in the training cohort

DCA, decision curve of analysis; CIC, clinical impact curve
